# Supplementary material for: The Effect of Varying Interval Definitions on the Prevalence of SARS-CoV-2 Reinfections: A Retrospective Cross-Sectional Cohort Study
Source: Diagnostics (Basel). 2022 Mar 15;12(3):719. doi: 10.3390/diagnostics12030719 (PMC8947046; doi:10.3390/diagnostics12030719)
Supplement: Supplementary file 1 [file diagnostics-12-00719-s001.zip › diagnostics-1622677-supplementary.pdf]

SUPPLEMENTARY MATERIAL

Supplementary Table S1. Number of reinfections according to different definitions, and corresponding Cp-values of first and second positive test.

Weeks between first and second positive test	Number of reinfections	Median Cp-value [IQR] first positive test	Median Cp-value [IQR] second positive test
1	684	25.1 [7.4]	33 [5.4]
2	260	25.9 [7.5]	34.1 [6.1]
3	132	26 [7.7]	33.2 [7.6]
4	89	26.5 [8.5]	32.5 [8.1]
5	73	26.5 [7.9]	31.1 [9.4]
6	68	26.4 [7.7]	29.8 [8.8]
7	59	26.3 [7.7]	29.2 [8.3]
8	52	26.3 [8.8]	28.7 [8.1]
9	48	26 [8.6]	28.7 [7.3]
10	46	26 [8.1]	28.7 [7.7]
11	45	26.1 [7.7]	28.7 [7.8]
12	44	26.3 [8.0]	28.7 [7.1]
13	41	26.1 [6.8]	28.7 [6.7]
14	39	26.5 [7.8]	28.7 [6.5]
15	37	26.5 [8.8]	28.7 [6.5]
16	37	26.5 [8.8]	28.7 [6.5]
17	37	26.5 [8.8]	28.7 [6.5]
18	35	26.8 [8.3]	28.1 [6.0]
19	34	26.6 [8.2]	28 [6.1]
20	34	26.6 [8.2]	28 [6.1]
Data are presented as number (%), or median[IQR]. IQR=interquartile range.			
